# Quinine Exanthem

**Published:** 1878-04

**Authors:** 


QUININE EXANTHEM.—Prof. Herman Cohn, of Breslau, reports a case of quinine exanthem occurring in a patient who had the prodominal symptoms of glaucoma and for whom he ordered quinine to reduce tension. On the same evening, after having taken 0.15 gr. (2 grains) of the quinine, the patient was attacked with a heavy fever, puffiness of the entire face, a scarlet eruption upon the breast, abdomen and legs. In fact, the case presented the characters of scarlet fever. The author regards the case as of the same nature as the one by Prof. Heinrich Kobnre (*Berl. Klin. Wochenochr.*, 1877, No. 22, 23).

This was a case of a man who was supposed to have had three attacks of scarlet fever in a year, but Kobner showed that the cause each time was excessively small doses of quinine. The eruption occurred the first time after 0.225 (3½ grains) of sulphate of quinine, and under the continued use of 1.275 (19 grains) of quinine, the flush continued eight days and the desquamation six weeks, on the soles of the feet for nine weeks; the highest and most persistent fever and the deepest prostration. The second time after 0.15 (2 grains) of quinine, exanthem four days flush, desquamation from five days on, course shorter and milder. The third time after 0.1 (1½ grains) quinine, exanthem flush for two and a half days, desquamation beginning on the third day, ending in two weeks, the shortest and mildest course.

Kobner mentioned four such cases as having been reported in the *British Medical Journal*, 1869–70, and briefly mentions the case of a physician of Breslau, who had a scarlet rash with a three weeks' desquamation, after taking 1 gr. (15 grains) of quinine. Prof. Hirt claims that only blondes are attacked with quinine eruption. Kobner makes no mention of the color of the hair. Prof. Cohn's patient was a deep brunette. Most of the patients had formerly suffered from skin affections, and Prof. H. remarks that it would be well before ordering quinine to ask the patients if they had now and then had erysipelas.—*Cent.-Bl. f. praktische Angenheilkunde.*

